# Hospital sinks and healthcare-associated infection: ecology, transmission, surveillance and mitigation

**DOI:** 10.1016/j.ebiom.2026.106415

**Published:** 2026-08-11

**Authors:** Aoife Kearney, Kevin Chau, Shireen Kotay, Jessica Martin, Andrew Kirby, Amy J. Mathers, Nicole Stoesser

**Affiliations:** a Nuffield Department of Medicine, University of Oxford, Oxford, UK; b NIHR Health Protection Research Unit in Healthcare-associated infections and Antimicrobial Resistance, Nuffield Department of Medicine, University of Oxford, Oxford, UK; c School of Medicine, University of Virginia, Charlottesville, VA, USA; d Leeds Teaching Hospitals NHS Trust, Great George Street, Leeds, LS1 3EX, UK; e Leeds Institute of Medical Research. St James's University Hospital, University of Leeds, Level 8, Wellcome Trust Brenner Building, LS9 7TF, UK; f NIHR Oxford Biomedical Research Centre, University of Oxford, UK

**Keywords:** Healthcare-associated infection, Sinks, Sink traps, Antimicrobial resistance, Infection prevention and control

## Abstract

Hospital sinks are recognised polymicrobial reservoirs for multi-drug resistant organisms and have been implicated in patient transmission and outbreaks. Earlier studies on sink-associated microbes predominantly focused on specific species or resistance mechanisms (e.g. carbapenemases) using targeted microbiological methods. More recently, less selective approaches (e.g. metagenomic sequencing) have enabled broader characterisation of these microbial communities.

This review summarises current evidence describing hospital sink-trap microbiomes, examining ecological determinants, surveillance strategies and interventions aiming to mitigate transmission from these reservoirs. We discuss biotic and abiotic factors that shape microbial selection/persistence, assess approaches to managing these reservoirs to reduce patient risk, and highlight priorities for future research to inform evidence-based practice in healthcare settings.

## Introduction

Hospital sinks and their sink-traps (i.e. ‘p-traps’, ‘bottle traps’, ‘waste-traps’ or ‘u-bends’) are increasingly recognised as pathogen reservoirs contributing to healthcare-associated infections (HAI) and outbreaks.^1^^,^^2^ Sink-traps retain wastewater by design to prevent egress of sewer gases. Polymicrobial communities in this wastewater may exist in different states, for example as surface-associated biofilms adhering to pipework or as planktonic cells within the retained wastewater. Sink-trap microbiomes facilitate AMR gene (ARG) exchange within and between species, including ARGs encoding broad-spectrum antimicrobial resistance (AMR) mechanisms (e.g. carbapenemases), via mobile genetic elements (MGEs).^2^, ^3^, ^4^, ^5^, ^6^ Much of the literature in this area has focussed on Gram-negative bacteria, but exposure to other potential pathogens such as non-tuberculous mycobacterium and fungi can also be facilitated by wastewater environments. Microbial spread to patients is thought to be primarily due to biofilm growth from pipework onto drain/basin surfaces, where water from taps impacts these contaminated surfaces and generates microbe-laden drops of water or splatter.^7^^,^^8^ Contaminated splatter is not confined to the sink itself but also to the “sink splash zone”, an area extending approximately two metres outside of the sink-trap.^9^ The presence of patients and patient-care items within this zone, and frequent interactions with sinks by patients, healthcare workers and hospital visitors, facilitates transmission.

There is currently no evidence-based consensus on how to best mitigate transmission from these reservoirs. Strategies include changing sink-use practices, decontamination, disinfection, renovation of clinical areas, engineered and novel sink design changes, sink upgrades and/or sink removal. How these approaches influence the shaping of sink-trap microbiomes and mitigation of risk from these reservoirs remains poorly quantified. Different scientific considerations, including ecological perspectives, might offer new and potentially important insights to inform interventions and guidance.

This article presents a narrative review of the literature relating to microbial populations within hospital sinks and their associated pipework, in a microbiological and infection prevention and control (IPC) context. Firstly, we identify evidence of ecological drivers influencing the microbial and resistome composition of sink-trap microbiomes, considering both biotic and abiotic factors driving pathogen and AMR selection in these reservoirs. Secondly, we summarise the current evidence characterising microbial transmission pathways from sink-traps to patients, quantifying the clinical impact of these on patient colonisation and HAI. Thirdly, we consider current and emerging strategies relevant to the microbiological surveillance of hospital sink-traps. Finally, we appraise the challenges associated with mitigation and summarise currently available strategies, including recent innovations that may represent potential future intervention options.

## Search strategy and selection criteria

A search of PubMed was performed without filters on 9th July 2026 [search terms: (bacteria∗ OR contaminant∗ OR microb∗ OR biofilm) AND (pipe OR sink drain OR p-trap OR u-bend OR sink OR “opportunistic premise plumbing pathogens”) AND (healthcare OR nosocomial OR hospital)] returning 623 results (Supplemental Data S1). Forty-nine further full-texts were identified by reviewing reference lists of relevant articles, or were known to the co-authors. Following screening of all abstracts, 245 full texts were reviewed by a single researcher (A. Kearney). Primary research studies directly addressing a theme underpinning the stated aims of this literature review were prioritised for final inclusion in the text.

To capture emerging technologies for sink-related microbial surveillance and mitigation, a patent search of Lens.org was undertaken on 9th July 2026 [search terms: (“sink trap” OR u-bend OR p-trap OR pipe OR plumbing) AND (microb∗ OR bacteria∗ OR pathogen∗ OR antimicrob∗ OR antimicrobial AND resistan∗ OR AMR) AND (cleaning OR decontaminat∗ OR disinfect∗)]. Results were limited to patents filed in the preceding 24 months to focus on the most recent novel approaches in development to be described, with 670 (following duplicate removal) relevant applications returned within this timeframe (Supplemental Data S1).

## The ecology of the sink-trap microbiome and drivers of AMR and pathogen selection in these reservoirs

**Fig. 1:**
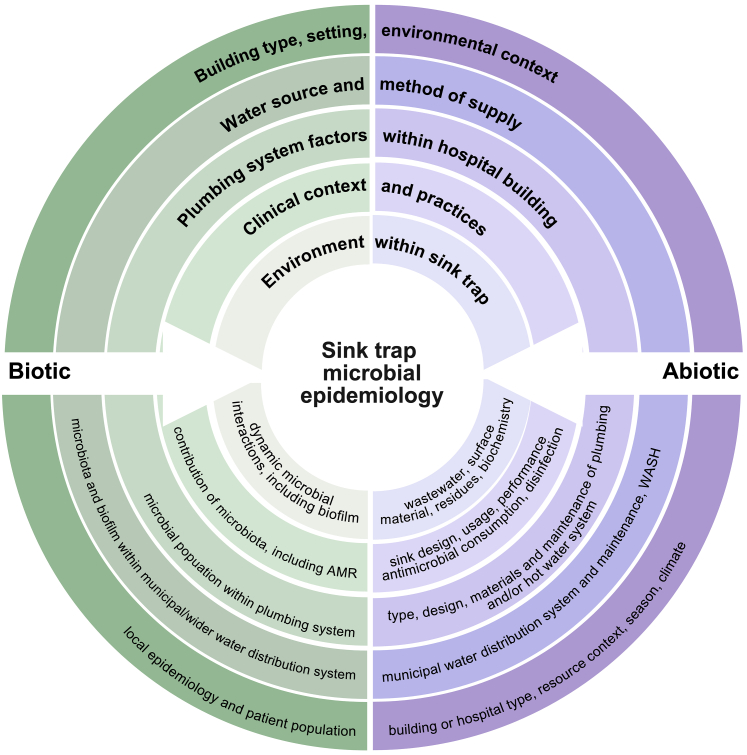
The microbial epidemiology of sink-traps is driven by various biotic and abiotic factors which may be static or dynamic. The environment directly within the sink-trap includes the microbial population (both planktonic, in the liquid within the trap, and biofilm adhered to the trap surface), the structure of the trap and residual substances (e.g. antimicrobials, disinfectants) contained within. Microbiota present within the trap arise both from sink-use (contributed by patients, healthcare workers etc.) and migration from within the wider water distribution and wastewater plumbing network. The ecology of the sink-trap is additionally modified by the design, placement, function and use of the sink. Each sink is connected to a wider plumbing system, which supplies water to the tap and removes waste from the trap to the wider system. This plumbing system is supplied by a local water source, and the quality, distribution method and WASH indicators relating to this varies. Created in BioRender. Kearney, A. (2026) https://BioRender.com/56iolxa.

Ecological studies typically describe ‘biotic’ and ‘abiotic’ factors influencing ecosystems. ‘Biotic’ variables describe living/once-living ecosystem components; ‘abiotic’ variables describe non-living chemical and/or physical elements influencing interactions and survival within ecosystems. Individual sinks represent discrete but interconnected micro-ecosystems within the larger hospital microbiome, in which many static or dynamic biotic and abiotic factors converge (Fig. 1).

### Biotic factors relevant to sink-trap ecology

Sink-traps are invariably microbiologically colonised, even if newly installed or immediately post-disinfection.^10^ Dynamic interactions between these microbial species (including bacteria, bacteriophages and eukaryotes such as fungi) contribute to the highly varied communities observed amongst sink-traps in the same setting,^3^^,^^11^ or within the same sink-trap over time.^10^^,^^12^ Microbial turnover and growth may drive marked changes in biotic factors within sink-traps over short timescales.

Microbial sources include tap water,^13^, ^14^, ^15^ handwashing (contributing organic matter e.g. skin,^16^ oils, urine, faeces^17^) and patient seeding either through expected sink use^18^ or through direct disposal of patient waste.^19^ Though many healthcare facilities mandate that sinks are used only for handwashing, a study observing thousands of healthcare worker/visitor/patient and handwashing sink interactions demonstrated that handwashing comprises a small amount (17%) of sink–person interactions.^19^ Retrograde colonisation of microbes from the wastewater system distal to the sink-trap may also occur, either from blocked or impaired drainage, or through biofilm-supported microbial migration along wastewater pipework. This connection to the wider hospital wastewater system may support microbial persistence and horizontal gene exchange within sink-trap environments.^5^^,^^20^ Microbial inputs to the sink-trap can therefore occur anterograde at the sink/user interface, or retrograde from the wider wastewater system into which the sink-trap is integrated.

Selection pressures, such as the presence of disinfectants or antibiotic residues in sink-traps^21^^,^^22^ (arising from clinical disposal or patient excreta), may influence microbial interactions. Co-operative interactions, for example degradation of the antibiotic carbenicillin by *Bacillus* spp. facilitating expansion of a mock sink community, have been described in-vitro.^23^ Other interactions may be competitive, as shown in a study investigating in-vitro competition between *Pseudomonas aeruginosa* and *Klebsiella pneumoniae* under conditions of nutrient limitation.^24^ On minimal media, both organisms attempted to scavenge iron by producing siderophores, but *P. aeruginosa* additionally produced a biosurfactant which prevented the non-motile *K. pneumoniae* from interacting with the nutrient medium, which was not observed when the medium was iron-enriched. Under dynamic conditions these microbial interactions may vary, potentially activated or deactivated by quorum sensing, and shifting from co-operative to competitive under fluctuating environmental conditions, selection pressures and nutrient availability.^25^

Tap water, even if of drinking quality, is not sterile.^14^ Microbial inputs to sink-traps through water supplies can change over time, even from highly engineered and well-maintained distribution systems. In settings with less secure access to clean water, this can pose further challenges. As an example, a cross-sectional study of water, sanitation and hygiene (WASH) indicators in Pakistan observed varied methods of water distribution to healthcare facilities including off-site piping, a borehole on the hospital grounds or by tanker truck.^26^ The authors further observed an association between the distribution type and carbapenem-resistant isolate detection in sink-traps.^26^ Factors related to water supply outside hospitals (e.g. municipal water treatment)^15^ or inside hospitals (e.g. point-of-entry^27^ or point-of-use water filters^28^) likely play a role in shaping sink-trap communities.

### Abiotic factors contributing to sink-trap ecology

Plumbing system factors may impact sink-trap communities. Contamination can occur during construction or renovation, and plumbing components are not sterile at the point of installation.^29^ A recent study observed *P. aeruginosa* prevalence was significantly higher in buildings with older plumbing systems, and in those with electric or solar hot water heating systems compared to gas; no significant differences were observed between buildings with or without hot water storage.^14^ Plumbing system materials may also be influential. In one in-vitro study designed to mimic hot water plumbing systems, three pipe materials (PVC, copper, iron) were inoculated with *P. aeruginosa* and *Acinetobacter baumannii*,^30^ and relative growth under different disinfectant conditions was investigated using metagenomics. Pipe material shaped taxonomic distributions and resistomes independently from, but also interactively with, disinfectants with certain combinations enriching for ARGs in pathogenic species. In contrast, an investigation of bacterial communities on ex-situ pipe materials (PVC, asbestos cement, cast iron) demonstrated similar alpha- (within community) and beta (between community)-diversity when water supply was uniform.^31^

Sink location^4^^,^^32^ may also influence microbial epidemiology. In a targeted study, CPE detection was associated with samples from sinks in direct proximity to toilets within patient rooms, compared to samples from sinks not adjacent to toilets; the authors proposed this may have been due to droplet dispersal during flushing or toilet-sink migration via pipework,^33^ or perhaps following hand hygiene after using the adjacent toilet. Sink location may also determine usage frequency. In a recent study, user-reported sink use was 15-fold higher in hallway sinks compared to sinks within single-bed patient rooms, and higher bacterial abundance (both 16s rRNA gene culture-based abundance) was observed in the hallway sinks.^13^

Sink use frequency and water flow rates can impact sink-trap environments. This was observed in a study where first-flush samples from handwash sinks and showers were collected in an Australian hospital, alongside temperature and flow rate data and estimated alpha- and beta-diversity using 16s rRNA sequencing.^15^ Flow rate influenced microbial community structure; species evenness was higher in under lower flow, with authors suggesting this represented greater stability and robustness of the microbial population under these conditions. In the aforementioned study where bacterial counts and certain taxa (i.e. *Pseudomonas* and *Bacteroides*) were significantly enriched in hallway sinks versus patient rooms, the authors proposed that more frequent usage (i.e. altering the flow rate and destabilising the microbiome) might benefit certain species by increasing sink-trap moisture and temperature, and by contributing nutrients and organic matter.^13^ Flow rate may additionally be influenced by sink design.

Sink-trap biochemistry (including pH, water hardness, alkalinity, presence of metals or oxidising agents) is not uniform even at ward-level,^22^ and plays a major role in shaping microbial communities (pre-print^34^). Chemical disinfection may induce rapid but transient shifts in community structure,^35^ and may select for pathogens and drug resistance^11^^,^^12^^,^^30^ or stimulate horizontal gene transfer (HGT), potentially promoting AMR gene dissemination.^36^ Other selection pressures include antimicrobial residues^21^^,^^22^–in a UK-based study which included 287 sink-trap aspirates from 29 hospitals, antimicrobial residues were detected in 33% of samples,^22^ and were associated with increased pathogen and ARG abundance (pre-print^34^). Nutrient-rich substances (e.g. enteral or parenteral feeding products, intravenous fluids/medication, and/or discarded beverages) which can promote bacterial growth^37^ are frequently disposed of via hospital sinks.^19^^,^^38^

Ecological determinants of sink trap microbiomes may influence microbial epidemiology, population structure and risk of transmission from these sites. The efficacy of mitigation strategies may also be determined or influenced by these factors. Reporting of these drivers in future intervention studies, where possible, may improve transferability and enhance interpretation of findings.

## Evidence in support of routes of pathogen transmission from sinks to patients

**Fig. 2:**
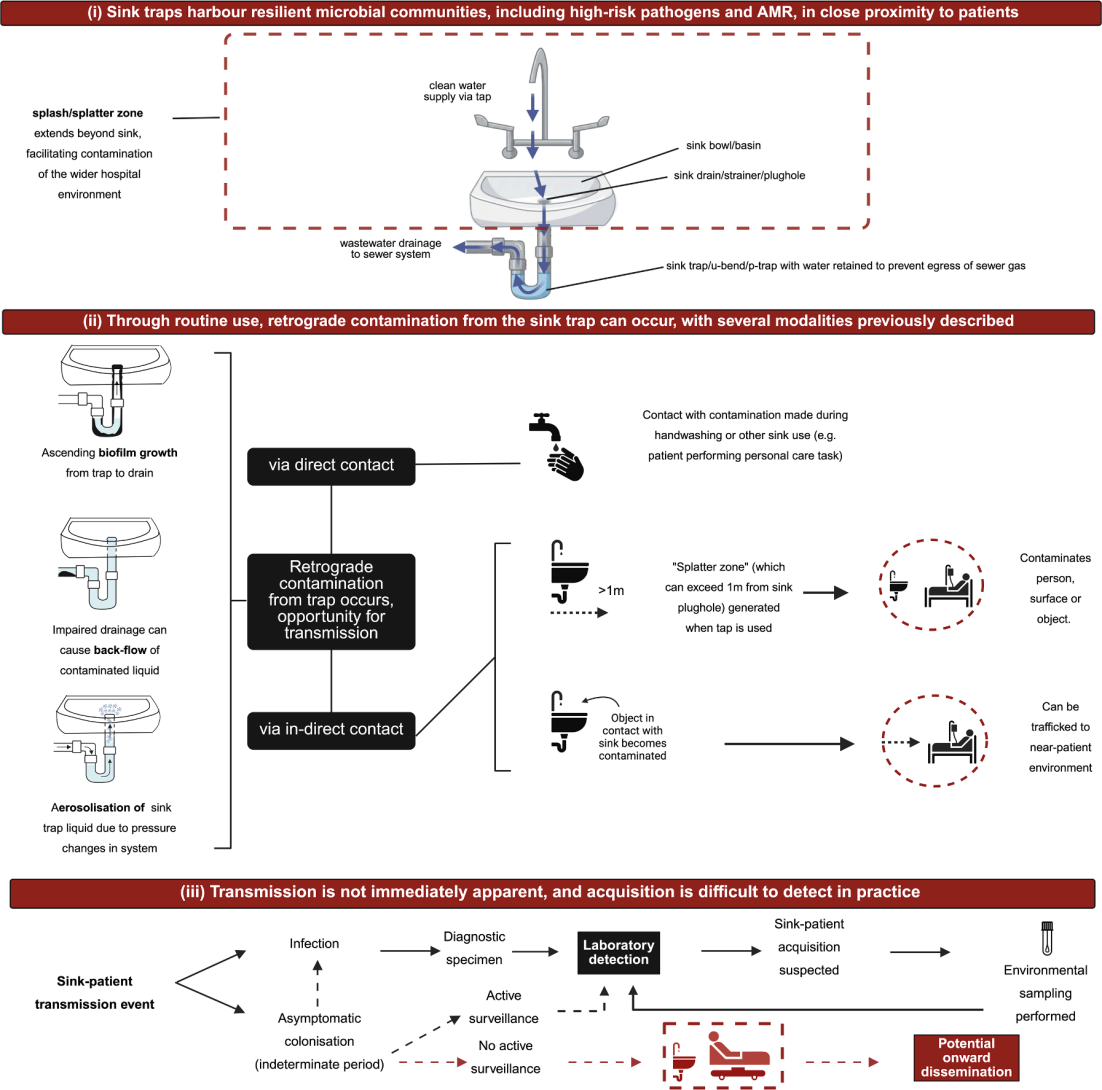
Several modalities underpinning sink-patient transmission of microbes have been described. This typically is thought to involve contamination of the sink drain or basin, which is then dispersed to a sink-user, surrounding surface or object when the tap is used. Identifying these transmission events in real-time is not possible. While transmission can result in infection, it can also lead to asymptomatic colonisation (± subsequent infection). Transmission may therefore be identified in a diagnostic specimen processed in a microbiology laboratory, or through a programme of active surveillance. In the absence of paired intensive clinical and environmental sampling, linking patient acquisition to environmental reservoirs is not possible. Created in BioRender. Kearney, A. (2026) https://BioRender.com/j7rofhx.

As non-sterile elements of a wastewater drainage system, sink traps will consistently harbour microbial communities. As patients do not interact with these sites directly, several studies have set out to describe how transmission from sinks to patients is understood to occur mechanistically (Fig. 2).^7^, ^8^, ^9^^,^^39^, ^40^, ^41^, ^42^ Dispersal through splatter of contaminated droplets is supported by several experimental evaluations. During a *P. aeruginosa* outbreak investigation between 2004 and 2006, a fluorescent marker was instilled within the sink-trap. The marker was dispersed from within the trap to the sink basin and surrounding sites (>1 m from the sink plughole) during normal sink-use^39^- emphasising the importance of sink design and placement in clinical settings. Contamination of the sink splash zone should be assumed to occur during routine sink operation.^9^ Dispersal was further investigated in another experimental study using green-fluorescent protein (GFP)-expressing *Escherichia coli* instilled in sink-traps.^7^ Ascending growth was observed from the sink-trap during simulated use, which extended to cover the strainer over a period of seven days. Strainer colonisation resulted in extensive droplet-mediated contamination of the sink and surrounding sites when the tap was used^7^; dispersal increased with higher flow rates.^43^ This splash or splatter puts patients and/or equipment within a 1–2 m zone at high risk of contamination,^39^ and patients are often cared for within this zone.^9^^,^^40^ Contamination may increase in cases where sink function is impaired, such as delayed drainage caused by limescale, biofilm build-up and/or blockages with clinical products such as paper towels.^41^^,^^43^ Transient positive air pressures within the wastewater system, which can occur independently of sink use, may also result in sink-trap-derived basin contamination via aerosols.^42^ Agitation within the wider wastewater system, due to air currents occurring during normal use or arising from maintenance, treatment or changes to functional performance of the system (either in terms of blockages or resulting from maintenance interventions downstream of the sinks) may influence how sink contamination occurs.^42^

Splatter/droplets could contaminate sink-users (e.g. during handwashing or cleaning), a surrounding surface, or piece of medical/other equipment which may be stored close to the sink.^8^^,^^9^^,^^39^ Several studies have identified items are commonly placed on/near sinks (12–43% of observations).^19^^,^^22^ Contamination may occur in sinks away from the patient bedspace (e.g. in kitchens or storage areas with sinks) and then be imported to the near-patient environment on contaminated fomites. Sink-to-bed ratios may be highest amongst the most vulnerable patient populations, such as critical care or haematology/oncology wards, where patients are commonly in single-occupancy rooms,^22^ with resulting higher patient:sink ratios. How patients interact with sinks either directly or with healthcare worker assistance, is not well-characterised. Patient practices may not align with intended sink function, for example patients may undertake sink-related personal care activities (e.g. bathing, oral hygiene) using the sink closest to their bedspace (which may be a designated staff handwashing sink). Healthcare workers incorporate sink use into multiple workflows, and sink use is not limited to handwashing only.^19^

Sink-to-patient transmission is likely multifactorial, and may depend on converging microbial, patient and wider contextual factors (both clinical and behavioural).^18^^,^^26^^,^^44^ While transmission likely carries greater risks for some patients than others (e.g. some patients have enhanced vulnerability to opportunistic infections associated with plumbing systems), harm can occur to all patient groups via this transmission pathway. Though much of the literature describes enhanced care contexts in acute hospitals,^1^ risk may also exist in long-term care facilities and in residential homes.^14^ Importantly, with patients that move between hospital and community settings, increases in ambulatory care delivery, and colonisation being a pre-cursor to infection for many pathogens, the contribution of sink-traps in residential settings to infection may be increasingly relevant, with at least one study demonstrating a higher prevalence of some potential pathogens in household versus hospital sink-traps.^14^ It is not known whether sink-traps in residential/long-term care settings pose similar risks of transmission as those in healthcare settings, or how this may relate to community-onset or hospital-onset but community-acquired infections. Healthcare system context is also likely influential; in many settings, challenges related to water, sanitation and hygiene (WASH) infrastructure and services are more pronounced, due to disparities in resources.^26^

The threshold of microbiological contamination of sink-traps/sinks associated with risks to patients is unknown; there is no currently defined ‘acceptable’ standard of contamination (comparable to a minimum infectious dose or water quality metrics for bathing water sites)^1^ or universally accepted healthcare building standards to minimise risk. Even if such a threshold was established, sink exposure does not consistently result in patient colonisation or infection, and there are likely microbial, host, sink design and use factors that may be protective or increase colonisation/infection risk.^18^^,^^44^ Efforts to capture transmission events are complicated by indeterminate periods of asymptomatic colonisation, and the complexities of within-niche colonisation diversity, which can go undetected in the absence of intensive active surveillance; however even intensive surveillance may not pick up all transmission instances. Hospital policies, mitigation strategies and local epidemiology may modulate transmission, but to what extent these different variables influence sink-related HAI risk is not yet clear.

## Quantifying the contribution of sink-traps to patient colonisation and healthcare-associated infection

**Fig. 3:**
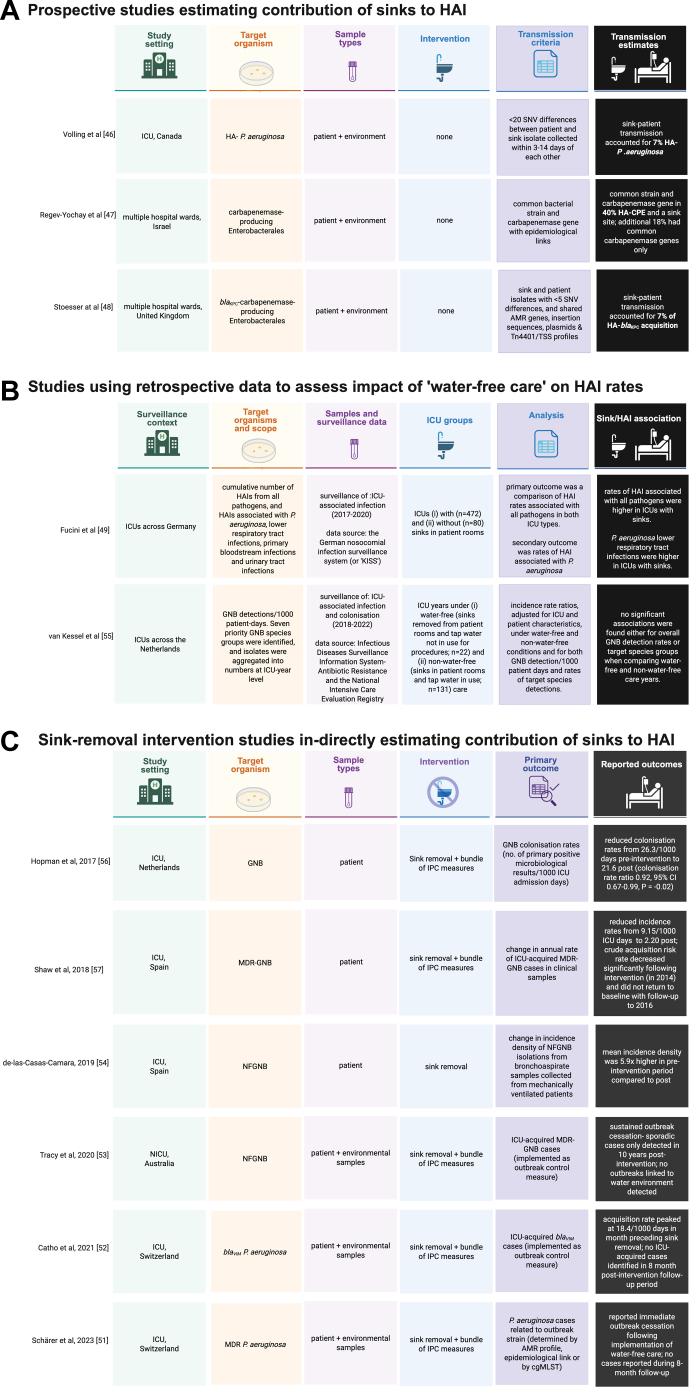
Different approaches have been undertaken to evaluate the relative contribution of sink-associated transmission to HAI. (A) Prospective studies have described intensive sampling of environmental sites alongside active surveillance of patients. Pre-set transmission criteria are used (typically using genomics-based thresholds) to establish whether sink-patient transmission has occurred. (B) Retrospective analyses of microbiological data reported to national surveillance systems. Incidence rates in ICUs where water-free care has been implemented are compared to non-water free settings. (C) By removing sinks and implementing water-free care, the contribution of sinks to HAI can be indirectly inferred. Intervention studies which have undertaken this approach have however typically involved a bundle of IPC interventions. Created in BioRender. Kearney, A. (2026) https://BioRender.com/e4dh3vh.

The relevance of sink-trap reservoirs to HAI outbreaks is well-described.^1^^,^^45^ However, reports have predominantly focused on specific bacterial targets (e.g. *Pseudomonas*, Enterobacterales) or AMR mechanisms (e.g. carbapenemases). While many studies detail spatial and temporal co-occurrence of patient and sink isolates, most are unable to quantify directionality or patient risk.^1^ This was highlighted in a systematic review in 2021 which used a causality tool to assess the quality of evidence supporting transmission of hospital-acquired Gammaproteobacteria (including clinically-important Enterobacterales and *Pseudomonas*) from sink drainage systems to patients across 52 eligible studies.^1^ The authors concluded that there was sufficient evidence that sink drainage systems are nosocomial reservoirs of healthcare-associated Gammaproteobacteria, but quantifying the attributable fraction of HAI was not possible. Since this review several studies making progress toward this aim are worth highlighting (Fig. 3A).

Healthcare-associated *P. aeruginosa* epidemiology was investigated in a prospective cohort study across six Canadian ICUs from 2018–2019.^46^ Active surveillance of patient colonisation using rectal screening was performed, alongside diagnostic sampling for infection. Environmental sampling of sinks used by healthcare workers (tap, sink-traps, air sampling during sink use) was undertaken on seven occasions. A genomic threshold of <20 single nucleotide variants (SNVs) between sink and patient isolates defined genetic relatedness, with sink-to-patient transmission defined as identification of a sink isolate 3–14 days prior to a genetically-related patient isolate. Using these criteria, sink-to-patient transmission accounted for an estimated 7% of healthcare-associated *P. aeruginosa*, with the authors noting this may be an under-estimate as sampling was not performed on approximately 40% of sinks, and respiratory tract colonisation (potentially occurring independently of rectal colonisation) was not surveyed.

Other studies have set out to quantify the contribution of sinks to the spread of CPEs/carbapenemase genes. One study analysed longitudinal samples over a 2-year period (2017–2019) investigating sink-patient CPE dynamics.^47^ Sink-to-patient transmission was thought to be a factor in up to 58% of 318 CPE acquisitions: 40% where both the species and carbapenemase gene were concordant between sink and patient samples, and in a further 18% where a common carbapenemase gene was observed between sink and patient samples. To establish directionality, temporal relationships between the linked samples were investigated. In 20 cases, the sink isolate was recovered prior to that from the patient, so a sink-to-patient transmission event was assumed. However, the authors noted that in most cases patient isolates were recovered prior to commencing environmental screening and directionality of transmission could not be clarified.

Another study sampled 1247 patients (including rectal swabs and diagnostic specimens) and 349 wastewater sites (n = 4488 environmental samples, 200 of which were sink drains) for *bla*_KPC_ Enterobacterales across six wards in a UK hospital over a 12-month period.^48^ Temporal relationships were evaluated for cases where patient and sink isolates differed by ≤5  SNVs and shared AMR gene, insertion sequence, plasmid and Tn*4401*/target site sequence (TSS) profiles. Twenty-nine genomically-supported transmission events were identified using these stringent criteria. Temporal dynamics supported environment-to-patient transmission in 7% of cases, patient-to-patient and patient-to-environment transmission in 45% of cases each, and environment-to-environment transmission in 3% of cases. Although this study focused on drug-resistant isolates, it indicates that similar routes of transmission are likely to be occurring for strains that are generally less of an IPC target, including more susceptible Enterobacterales.

In addition to prospective sampling studies which set out to define microbial/gene-based transmission events, observational and interventional studies have also sought to quantify the risk from sinks to patients (Fig. 3). In a study combining data from the German HAI surveillance system (2017–2020) with details of infrastructure from 597 ICUs,^49^ ICUs were split into two groups: those with sinks in patient rooms (n = 472) and those without (n = 80). Multivariable analysis demonstrated higher incidence of HAIs associated with all pathogens in the ICUs with sinks, and the presence of a sink within a patient room was an independent risk factor for the development of HAI (IRR: 1.21 [95% CI: 1.01–1.45]; p = 0.019). The authors highlight a potential confounder in that ICUs without sinks in patient rooms (whether single- or multiple-beds) were of newer construction (more often built after 2010) which may mask the impact of systematic, temporally driven differences in hospital design and/or IPC/cleaning practice that were not ascertained. However, of note, construction era was considered in the initial multivariable model but not retained in the model following stepwise selection.

Some studies have indirectly demonstrated the contribution of sinks to patient colonisation/HAI by removing sinks and implementing ‘water-free’ care in single centres. These have typically been undertaken in ICU settings in response to bacterial outbreaks (including associated with AMR), and were summarised in a recent systematic review^50^ which identified seven studies of quasi-experimental design conducted across 16 ICUs in total. Most described bundled interventions and considered different outcomes. Some identified outbreak termination as the primary outcome,^51^, ^52^, ^53^ and did not observe cases associated with the outbreak following sink removal (although with variable follow-up durations). One prospective study implemented sink removal alongside one other practice change (ceasing same-patient re-use of masks for nebulisation), and observed reduced mean incidence of non-fermenting Gram-negative bacilli isolation per 1000 invasive mechanical ventilation days pre- (11.28) and post-intervention (1.90).^54^ In addition, fold decreases in antibiotic use days (1.29), patient isolation days (1.51) and ‘multi-resistant bacteria’ days (1.84) were observed. In the remaining studies within this review, all with bundled interventions, decreased Gram-negative bacilli colonisation and/or HAIs were reported when sinks were removed.

These findings contrast with a more recently published study from the Netherlands using retrospective surveillance data collected between 2018 and 2022.^55^ Detections of Gram-negative bacilli (GNB; encompassing both infection and colonisation) per 1000 bed-days were compared between ‘water-free’ ICU years (tap water not used for patient care procedures and sinks completely removed from patient rooms; n = 22) and ‘non-water-free’ ICU years (tap water used and sinks present; n = 131). No association was found between water-free care and GNB detection rates under non-outbreak conditions. The authors of the more recent Netherlands-based analysis highlighted factors which may explain the different conclusions between their study and the earlier work.^49^^,^^51^, ^52^, ^53^, ^54^^,^^56^^,^^57^ These include differences in outcomes (e.g. use of a composite outcome of infection and colonisation, rather than infection or colonisation separately), differences in the microbiological focus (e.g. total GNB, non-fermenting GNB, MDR-GNB, *P. aeruginosa* or total HAI) and the widespread use of selective digestive decontamination in the Netherlands (uncommon elsewhere).

## Challenges in the microbiological surveillance of sink traps

**Fig. 4:**
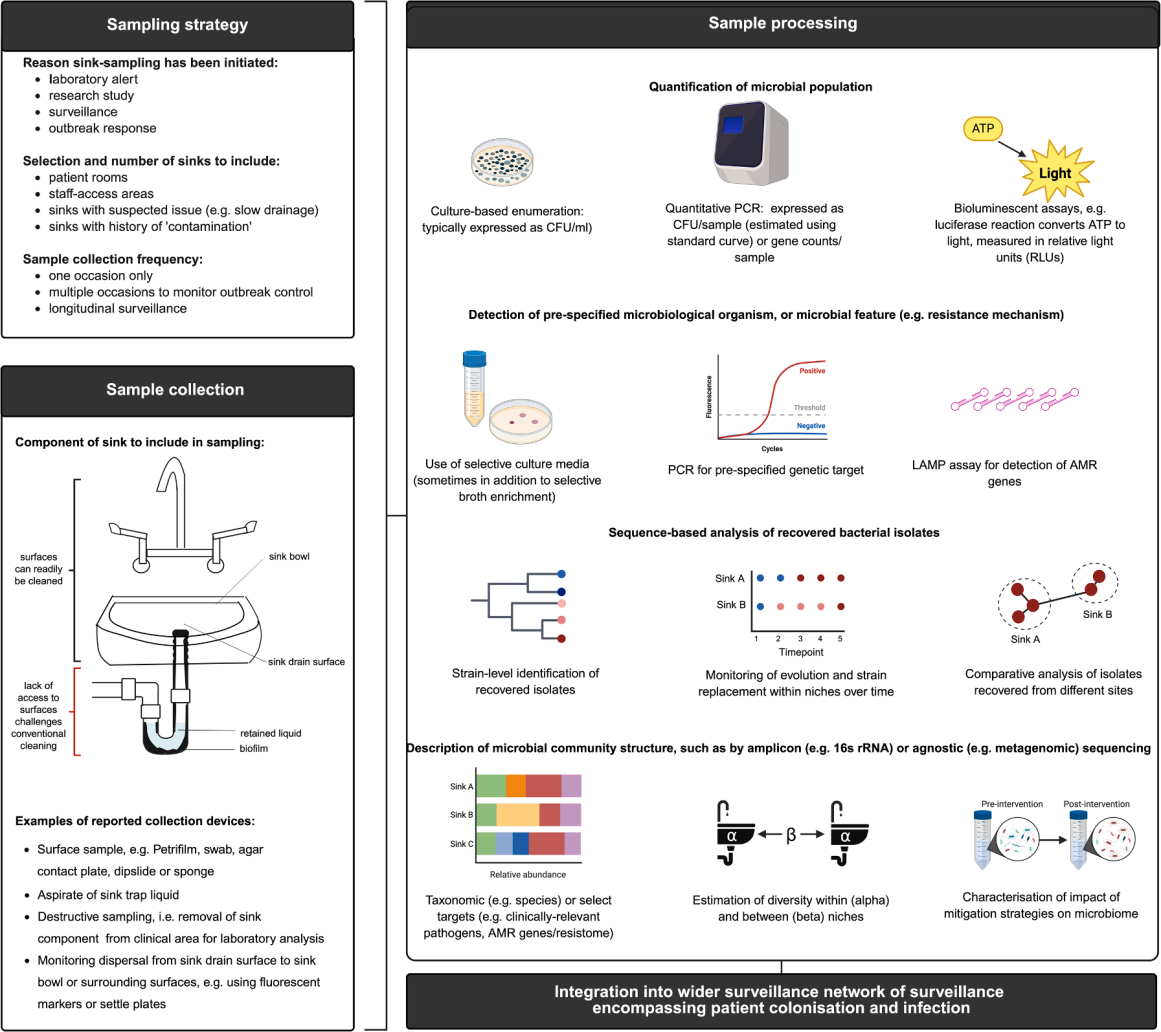
Sink sampling and monitoring strategies. There remains no consensus about how environmental surveillance and monitoring of sinks should be performed, and multiple approaches have been described in outbreak reports, prospective studies and in the evaluation of interventions. Sampling strategy (in terms of study aim, sink selection and sampling frequency) is varied; there are multiple methods describing the part of the sink to include in sampling. Microbiological processing can be undertaken using culture-based or molecular methods. Results can be expressed in terms of the number of bacteria present in a sample or whether a specific microbial target is present or not. Sequence-based analysis can facilitate strain-level identification and tracking within the hospital environment or can describe the community structure within and between sinks. Created in BioRender. Kearney, A. (2026) https://BioRender.com/59v0vax.

Sink-trap sampling practices vary substantially, including choice of sink-site (e.g. sink-trap versus sink-drain), device (e.g. aspirate versus swab) and processing-method (e.g. broth enrichment versus direct processing of samples, selective culture-based processing versus agnostic metagenomic sequencing) (Fig. 4). Currently, there is no consensus on optimal methods for environmental microbiological surveillance of sink-associated sites in healthcare facilities, and this surveillance is often responsive (e.g. in an outbreak). Culture-based methods can be time-consuming, particularly for some pathogens such as non-tuberculosis mycobacteria, which can require extended laboratory processes.^27^

Targeted approaches often dichotomise findings, classifying sites as “contaminated” or “not contaminated” with specific bacterial targets (e.g. *P*. *aeruginosa*) or AMR mechanisms (e.g. carbapenemase). Although microbial burdens differ based on the sink-site sampled^10^^,^^13^ and over short periods of time,^12^^,^^58^ quantitative and longitudinal reporting is uncommon, and endpoints used in intervention studies or outbreak investigations are not typically designed to capture this nuance. The wider role of the sink microbiome in facilitating or countering pathogen/AMR gene proliferation (e.g. by providing colonisation resistance) has historically been overlooked, but there is increasing awareness that these microbial communities are subject to multiple influences and potentially rapid and dynamic shifts.^12^ As such, broader approaches which attempt to quantify changing microbial loads (ideally rapidly or near real-time), and/or characterise the wider microbial community in addition to specific targets, have the potential to reveal new insights that could guide sustainable mitigation strategies such as microbiome manipulation. Technologies such as metagenomic sequencing may enhance our understanding of the role of the healthcare environment in pathogen transmission, AMR emergence and HAI, but remain challenging to implement, given the lack of standardised workflows, the fact that different workflows can generate different results, and the requirement for specific computational infrastructure (e.g. access to servers, storage and networking to process, manage and store sequence-associated data) and bioinformatics expertise.^59^

Previous studies have quantitatively compared contamination at different sink sites, observing higher microbial concentrations in surfaces distal to the sink drain (i.e. in the waste pipework).^10^^,^^13^ Significant differences in community structure, composition and beta-diversity between within-sink-trap swab samples and those collected from pipework immediately distal to the sink drain have also been observed.^10^^,^^16^

Most studies do not sample longitudinally so current understanding of evolution and population dynamics in sink-trap microbiomes over time is limited. As sinks exist within dynamic healthcare contexts it is unsurprising that microbial communities fluctuate.^13^^,^^48^ As an example, focussing on *bla*_KPC_ Enterobacterales, one study observed that hospital wastewater sites including sink-traps yielded a median of 3 (range: 1–17, interquartile range: 2–5) genomically-characterised strains during a 6–12 month sampling period; in 77% (41/53) of longitudinally sampled sites that were positive for *bla*_KPC_ Enterobacterales, strain composition changed within the same site during the study.

Furthermore, within-sample strain-level diversity is not typically reflected in current sampling strategies. In the same study multiple colonies from positive samples were sequenced to determine within-niche diversity and evaluate evolution and transmission modalities.^48^ In 24.4% (78/319) of *bla*_KPC_-positive environmental samples, >1 strain was recovered. Markers of transposition frequency of the *bla*_KPC_-associated transposon, Tn*4401*, were more common in environmental isolates (94%, 66/70) compared to those collected from patients (17%, 12/70; Fisher's exact test, P < 0.001). This suggests conditions within environmental sites (including sinks-traps) supported horizontal gene transfer of *bla*_KPC_ to different species and strains. Incomplete characterisation of diversity and the absence of longitudinal surveillance may contribute to improper inference regarding risk and transmission.

It is unclear whether biofilm or planktonic cells (or both) are most relevant in terms of clinical risk. Several studies describe different collection and analysis methods to investigate sink-trap biofilms and the effects of interventions on biofilm structure. Some methods are performed *in situ,* such as swabbing the trap surface^13^ or agitating surface-associated biofilm with a catheter.^32^ Others involve sink-trap removal for laboratory-based assessment, including direct staining with crystal violet post-washing,^60^ or biofilm extraction through sonication,^61^ scraping^62^ or using sponge.^10^ Results may vary substantially depending on the method used. Standardised assays for laboratory-based biofilm analyses–which generally remove planktonic cells prior to analysis–exist but have not been specifically applied to sink-traps.^63^^,^^64^ Harmonising sampling strategies and developing transferable, reproducible protocols would improve the comparability and reliability of analyses, especially where removal or reduction of biofilm acts as a study endpoint.

Some studies have used alternative methods (often optimised for rapid turnaround) incorporating indirect measurement of bacterial loads to monitor sink contamination. Intracellular adenosine triphosphate (ATP) to estimate bacterial counts^12^^,^^65^ and loop-mediated isothermal amplification assays to detect specific bacterial targets^66^^,^^67^ or AMR mechanisms^68^ may offer rapid, cost-effective alternatives to culture-based processing, though evidence supporting their use is still being generated. In many settings, including LMICs, microbiology laboratory access is not widely available for clinical specimens,^69^^,^^70^ and capacity to process additional samples from environmental surveillance campaigns is negligible. Even in high-income countries, access to environmental sampling laboratories and capacity for processing is atypical. A recent review of sink-related microbial surveillance in LMICs proposed a tiered strategy including routine longitudinal monitoring (using routine selective culture with quantification of total burden with qPCR), reflexive targeted PCR (when set contamination thresholds are exceeded) and periodic metagenomic sequencing, notwithstanding challenging deployment capacity and the issues of access to sufficient resource.^71^ Rapid, culture-independent point-of-care assays to monitor contamination may enhance real-time environmental stewardship and IPC practice, especially if thresholds for acceptable levels of contamination are agreed which could guide use of resources.

Given sinks are widespread in healthcare settings, universal monitoring and mitigation measures would require major investment; prioritising resource allocation with other competing healthcare priorities is challenging. One study used a “super-spreader” model to identify a subset of high-transmission risk sinks to prioritise for intervention.^72^ Interventions were prioritised for 30/102 (29%) sinks from which splatter (evidenced using sink performance, fluorescent gel and cobalt chloride water leak indication paper) was demonstrated.

Approaches requiring less laboratory processing than conventional culture-based enumeration such as the use of Petrifilm,^73^ dipslides,^74^ or placement of settle plates on sink-adjacent surfaces to monitor dispersal have been described.^8^^,^^41^^,^^75^ Markers of sink function, such as dispersal evidenced using fluorescent dye may also be beneficial and return results rapidly.^76^ A potential advantage of simple environmental monitoring methods is that they do not require significant microbiological expertise, so could be performed by non-specialist hospital staff with appropriate training (e.g. estates or cleaning teams). Integrated surveillance could promote shared data ownership and aid multi-disciplinary intervention. While these methods require further validation to associate thresholds with risk levels,^1^^,^^77^ there may be implementation opportunities that enhance environmental surveillance.

When considering the innovation landscape, we identified five patent applications (filed between 20/09/2025 and 09/07/2026) with claims applicable to sink-trap surveillance. One patent describes an in-pipe sensor producing infrared signals capable of alerting to changes in biofilm, microorganisms or biological contamination.^78^ The pipe segment additionally contains a sampling port/auto-sampler and ports to facilitate cleaning agent instillation. Other sensor technologies monitor disinfectant concentrations or water biochemistry, supporting dynamic feedback loops capable of maintaining optimal concentrations of electrochemically-activated water^79^ or hypochlorous acid.^80^ The patent search additionally identified several emerging diagnostic technologies that could potentially be adapted for sink-associated monitoring, including analyte-specific coffee-ring biosensors^81^ and microfluidic cell counters for CFU enumeration.^82^^,^^83^

Integration of sink monitoring into wider environment and patient-level surveillance in a way that is evidence-based, cost-effective and improves patient outcomes is yet to be determined. Conventional IPC-based microbial typing and surveillance has traditionally relied on strain tracking, which may be less appropriate where polymicrobial reservoirs are involved in transmission, and where HGT is relevant to risk.^5^^,^^48^ While more comprehensive assessments of sink-trap microbial communities may be helpful in guiding mitigation, resource-intensive methods may not be feasible for real-time surveillance. Consensus on standardised and widely-implementable surveillance approaches would support implementation of IPC interventions and facilitate evidence synthesis between studies.

## Infection prevention and control interventions to mitigate the risk of transmission from sink-traps in healthcare settings

**Fig. 5:**
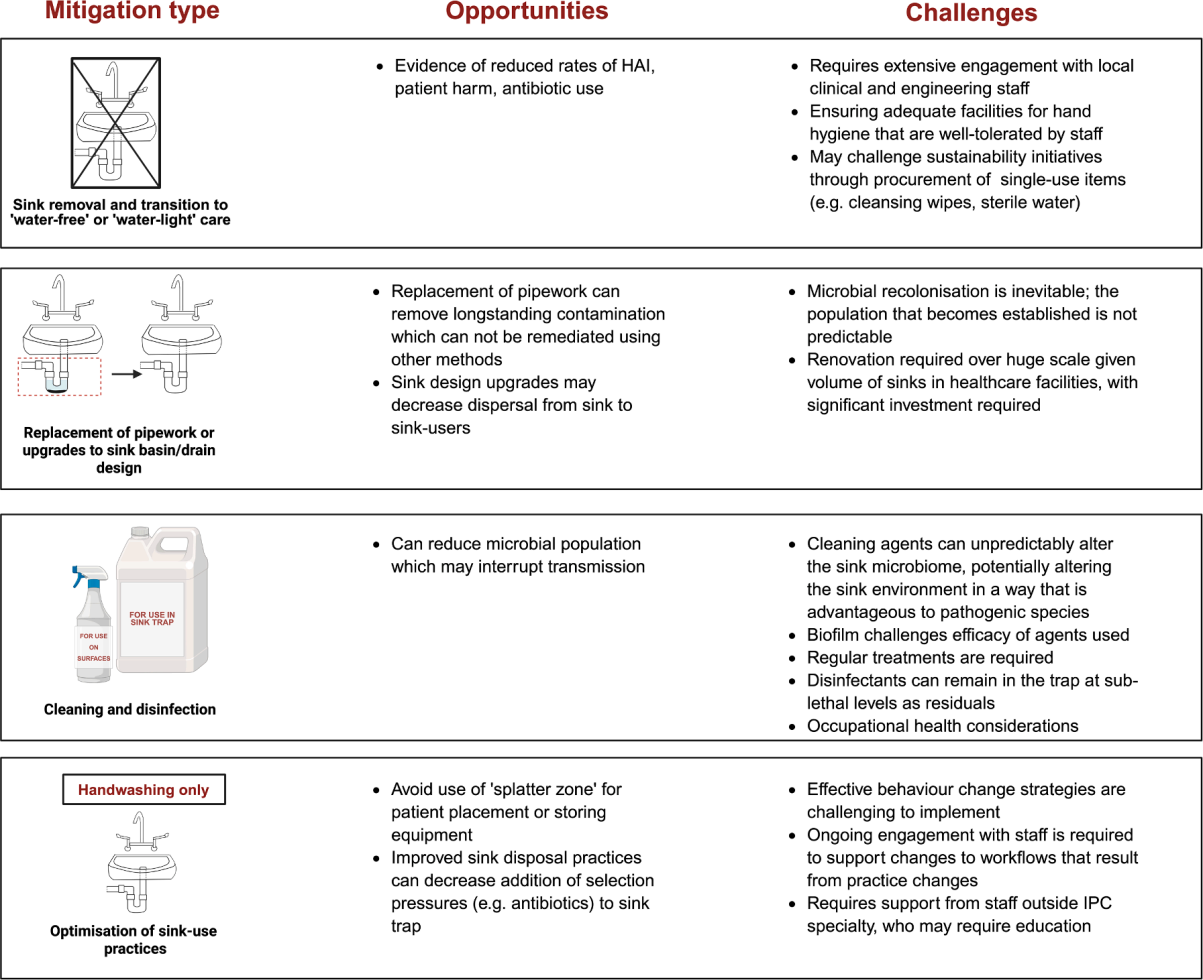
Sink risk mitigation strategies. Strategies described in the literature typically involve sink removal, replacement or upgrades of plumbing fixtures, mandating regular flushing regimens, cleaning or disinfection or attempt to modify sink-use behaviours. There remains no agreement in the literature on which approach is broadly implementable, feasible and evidence-based; each approach has distinct opportunities and potential advantages, though challenges have been reported for each. Created in BioRender. Kearney, A. (2026) https://BioRender.com/wu3pstu.

Sink interventions and study designs vary widely (Fig. 5). Study endpoints include eradication of specific microbiological targets (e.g. CPE) from sink samples or reduced rates among in-patients,^84^^,^^85^ reduced bacterial burden^12^^,^^58^^,^^65^^,^^86^ or characterising intervention-induced bacterial population changes.^12^^,^^58^^,^^65^^,^^87^ Despite several reports of target species ‘resurging’ post-intervention,^58^^,^^88^ follow-up durations differ.^50^ Interventions are typically bundled with other IPC strategies,^50^^,^^89^ making the contribution of the sink-orientated measures challenging to discern. Patient epidemiology differs, and sink-specific microbiomes can vary significantly even within the same hospital,^3^^,^^34^^,^^48^ limiting generalisability of findings.^89^ Cost-effectiveness is not typically evaluated. A recent systematic review identified the need for prospective studies in non-outbreak settings which integrate sequence-based analysis, and recommended reporting of confounders including antibiotic consumption and comorbidities.^89^

Several studies describe reduced colonisation/HAI rates following sink removal.^51^, ^52^, ^53^, ^54^^,^^56^^,^^57^^,^^90^ Interventions which remove sinks and other plumbing fixtures are sometimes referred to as ‘water-less’ or ‘water-free’ care (i.e. where patient interaction with wastewater systems such as sinks is essentially eradicated) or ‘water-light’ (where patient-plumbing interactions are curtailed through practice changes, sometimes with modifications to wastewater infrastructure) care. This concept was reviewed in 2024 by Inkster and colleagues,^91^ who describe the aims of these strategies as two-fold: to reduce interactions with bacteria (i) associated with clean water distribution through taps (e.g. *Legionella* spp.) and (ii) associated with the wastewater system such as sink-traps and shower drains. Evidence and feasibility of these strategies beyond augmented care settings in high income settings is not yet established (earlier detailed in Fig. 3).^92^

Some implementation challenges associated with shifting towards water-free or water-light care are additionally worth highlighting. Procuring single-use bottled water or disposable wipes to replace tap water^51^, ^52^, ^53^^,^^56^^,^^57^ may conflict with sustainability initiatives.^93^ Such products carry IPC risks too and may harbour bacterial contamination–nosocomial outbreaks linked to cleansing wipes have been reported.^94^ Surveyed healthcare workers and engineers identified the need for unit-specific solutions, highlighting for example the inappropriateness of cleansing wipes in neonatal settings.^95^ Acceptability of water-free care for patients, both in terms of comfort and anxiety regarding hygiene is an important consideration, and is an under-researched topic. Engaged partnership with local clinical staff pre- and post-implementation has been emphasised previously.^96^ Enabling alternative hand hygiene is essential,^95^^,^^97^ though shifting practice from sink-use toward alcohol-based hand gel may be difficult to tolerate by healthcare workers. As an example, in a water-free NICU, 43.6% (44/101) experienced dermatological issues, and 10.9% (11/101) sought medical attention.^97^

Renovations in transition to water-free care may have unintended consequences. For example, four years after the initiation of water-free care in one ICU,^52^ an infestation of larvae was detected in the wastewater network.^98^ Drainage of dialysis effluent had been maintained via removable wall ports; pipes had been left in-situ and sealed off. Disrupted wastewater flow under water-free conditions led to this stagnation, creating an environment supporting larval growth and leading to infestation.^98^ While patient harm was not reported, resolution involved combined thermal and chemical disinfection.

Most in-patients are not cared for in water-free environments. Some studies have evaluated how sink infrastructure and design can be optimised to prevent dispersal of trap-associated microbiota, including by ensuring drains are offset from taps, by altering flow dynamics using basin fins and ensuring drainage is not impaired.^41^^,^^75^^,^^99^ Regular sink cleaning and removal of debris (e.g. hair build-up, small discarded items), primarily performed by designated cleaning staff (also sometimes referred to as environmental service workers, janitors or housekeepers) is therefore integral to risk mitigation, regardless of sink design, although it may be challenging. Adherence to product contact times when cleaning vertical sink-trap pipework is difficult; workarounds have included approaches such as controlled injection of disinfectant to the trap over a defined time period,^100^ temporary obstructions (such as the inflated balloon of a urinary catheter)^101^ or manual^102^^,^^103^ or automated^104^ valves capable of preventing drainage while a disinfectant is in-situ.^85^ Foamed disinfectants allow greater contact time than liquid, appearing to increase efficacy, though bacterial counts return to baseline within days even after repeated treatments.^105^, ^106^, ^107^

Disinfectant choice requires careful consideration. Impacts on microbial counts and populations can vary widely,^106^ even when developed specifically for use in hospital sinks^35^^,^^108^ or when the same product is being evaluated in different studies.^58^^,^^109^ Residual exposure, including at sub-inhibitory levels, has been shown to promote horizontal gene transfer (including ARG transmission).^110^, ^111^, ^112^ Selection of disinfectant/biocide-resistance mechanisms remains a risk.^113^, ^114^, ^115^ Diverse sink-trap microbiomes may respond differently to cleaning regimens, making generalisable solutions challenging. Conflicting reports on the efficacy of disinfectant-based cleaning, both in terms of the impact on the microbial population and in preventing transmission to patients, highlight the need to carefully balance risks and benefits.

Some recent studies have characterised the impacts of interventions on the microbial population within sink-traps. One study evaluated peracetic acid disinfection by enumerating intracellular ATP (as a proxy for total bacterial counts) in drain aspirates alongside metagenomic sequencing.^12^ Bacterial counts did not change significantly during the intervention. However, the microbial community structure was altered; *Pseudomonas* spp. abundance significantly increased, while *P. aeruginosa* abundance remained stable. A further study investigated the impact of a single application of a 3.13% hydrogen peroxide, 0.099% octanoic acid and 0.05% peroxyacetic acid disinfectant to 12 sinks in a US haematology ward (compared with seven untreated controls).^58^ Total culturable counts decreased by 2–3 log immediately post-disinfection, rebounding within four days. Following disinfection, growth on selective carbapenem-resistant media was significantly higher in treated sinks versus controls. Metagenomic analysis on a subset of samples showed disinfection significantly decreased alpha diversity and increased pathogen abundance. Disinfection appeared to select for taxa with chromosomally encoded resistance mechanisms, whereas MGE-encoded resistance decreased.

Genomics can provide additional insights on the impact of cleaning on specific strains/species of interest, as shown in a cross-over intervention study undertaken in two critical care units of a US hospital investigating strain evolution of *Stenotrophomonas maltophilia* and *P. aeruginosa* in response to sink-trap cleaning with foamed disinfectant.^87^ *P. aeruginosa* populations underwent strain replacement in intervention sinks in contrast to *S. maltophilia*, where strains persisted in both treated and control sinks. Enhanced resolution offered by sequence-based approaches can complement targeted microbial monitoring during prospective studies,^84^^,^^85^^,^^87^ and can give greater insight into the ecological effects of cleaning interventions.

Occupational health of cleaning staff must be considered.^116^^,^^117^ The US National Institute of Occupational Safety and Health undertook a health-hazard evaluation following staff-reported health concerns following introduction of primary surface cleaning using a sporicidal hydrogen peroxide, peracetic and acetic acid product.^118^ Though air samples found occupational limits were not exceeded by cleaning staff, self-reported symptoms in post-shift surveys revealed associations between disinfectant use and acute (cross-shift) and chronic (previous four weeks) eye, upper- and lower-airway symptoms. Additionally, cleaning staff may themselves be at risk of acquiring sink-associated microbiota during cleaning.^119^ Novel approaches to sink risk mitigation that also address risks to staff include self-disinfecting sink-traps,^108^^,^^120^, ^121^, ^122^ designs which limit splash-back^86^ or have modified surfaces with antibacterial nanocoatings.^123^

To circumvent challenges associated with managing established biofilm in sink traps, pipework is sometimes be replaced by hospital plumbing teams. However, recent evidence suggests that renovation may unfavourably alter biofilm community structures.^124^ In a study characterising microbial communities before and after replacement of six sink traps in an ICU, shifts toward abundance of Enterobacterales, including genera associated with both HAI and AMR, were observed in post-renovation biofilms whereas abundance of slow-growing typically aquatic genera decreased.^124^ These shifts were associated with increased resistome loads, including of clinically relevant AMR determinants.^124^

A recent review highlighted biological control using bacteriophage or probiotic agents as potential mitigation strategies.^125^ Biological control (‘biocontrol’) strategies seeking to promote a ‘healthy’ sink-trap microbiome that exhibits colonisation resistance against pathogens and limits AMR emergence have been proposed. Probiotic cleansers (containing *Bacillus* spores) demonstrated favourable surface contamination (decreased microbial and ARG counts, increased alpha-diversity) relative to conventional agents previously,^126^^,^^127^ though use in settings housing immunocompromised patients (e.g. haematology/oncology) requires careful risk assessment. Bacteriophage have been evaluated in-vitro, with reductions in planktonic and biofilm biomass observed.^128^ In reviewing the current literature, reduced sink-trap alpha-diversity has been significantly associated with both increased abundance of ARGs and clinically-relevant pathogens (pre-print ^34^). This may echo microbiome dysbiosis in humans where decreased diversity can signal reduced health and contribute to dysbiosis-associated colonisation or infection,^129^^,^^130^ and microbiota transplantation has been considered as treatment. There may therefore be opportunities to exploit microbiome engineering as an intervention, but further research is needed.^11^^,^^34^

Amongst patent applications with relevance to mitigation of contamination and/or microbial transmission from sink-traps, we identified 11 potentially relevant innovations filed between 12/09/2024 and 09/07/2026. The majority of these related to surface modification, either through surface modification, coatings or structural alterations that could potentially be applied to sink-traps.^131^, ^132^, ^133^, ^134^, ^135^, ^136^, ^137^, ^138^ Others described ultraviolet or ultrasonic disinfection methods,^139^^,^^140^ antimicrobial peptides^141^ or disinfectant products.^142^, ^143^, ^144^, ^145^, ^146^, ^147^

Consensus on optimal approaches to environmental monitoring, selection of study endpoints, and measurement of clinical impacts would substantially strengthen the evaluation of interventions. Establishing frameworks that enable evaluation initially in controlled laboratory settings and subsequently in adequately powered hospital-based studies is essential; current practices remain highly variable. Given the rapid expansion of the literature, maintaining up-to-date guidance is challenging. A “living guidance” model capable of rapidly incorporating emerging evidence may be more favourable, supporting evidence-based decision-making around risk mitigation.

Without a generalisable, low-risk, and cost-effective mitigation strategy, strengthening evidence-based safeguards and focussing effort in high-risk areas in the existing built environment is a pragmatic starting point, with a view to rolling out best practice strategies more widely. Examples include assessing splash zone risk and ensuring care items or patient bedspaces should not be located within these zones. Sink drainage and design should be optimised where possible (e.g. by preventing and rapidly addressing blockages) to reduce risk of patient-to-sink and sink-to-patient transmission of pathogens. Sinks should not routinely be used for disposal of liquid wastes which act as nutrients or selection pressures, including antimicrobials, and healthcare workers should be supported to make these adjustments. In partnership with facilities teams, safely removing sinks where clinically unnecessary may reduce risk, while rationalising the number and placement of remaining sinks may help ensure safer healthcare environments.

## Outstanding questions

The risk of sink-associated microbial transmission is currently being managed by IPC teams without a data-supported implementation pathway–a critical gap given the need to recognise and rectify issues rapidly. While there is now sufficient evidence that sinks are inherently colonised with microbes and can serve as reservoirs for high-risk and AMR pathogens, evidence on safe and effective mitigation is still needed. Adequately powered, prospective intervention studies are urgently needed to support clinical decision-making and guide cost-effective IPC practice across settings.

The ecological drivers of pathogen and ARG burden within sink trap microbiomes remain incompletely understood. Identifying microbial, genomic or functional signatures associated with increased transmission risk or dysbiosis could enable risk stratification and more targeted environmental stewardship in hospitals. Much of the focus to date has been on Gram-negative pathogens, but emerging evidence suggests that sink traps are also relevant for other potential pathogens, such as non-tuberculous mycobacteria and fungi, and this should be explored further. Water-free and water-minimising care models show promise, but scaling implementation beyond augmented care units will bring new challenges. Clinical plumbing interfaces–including sinks, showers and toilets–will remain ubiquitous in healthcare settings for the foreseeable future. Though essential, sink-related maintenance and hygiene practices must be carefully evaluated. Paradoxical selection for HAI- and AMR-associated microbes through cleaning and disinfection is an underappreciated risk that warrants explicit research attention. Patient and healthcare worker consultation will be essential to ensure interventions will be acceptable and sustainable in the long-term.

## Contributors

Conceptualisation: All authors; Data curation: AKe; Funding acquisition: NS; Investigation: AKe; Methodology: AKe, NS, AKi; Project administration: AKe, NS; Resources: AKe, NS; Supervision: NS; Validation: All authors; Visualisation: All authors; Writing—original draft: Ake, NS; Writing—review and editing: All authors.

All authors read and approved the final version of the manuscript.

## Declaration of interests

AKi has received financial support from the Healthcare Infection Society (United Kingdom) and National Institute for Health Research (United Kingdom), and has received consulting fees from Johnson & Johnson. AKi chairs the Research Grants Committee of the Healthcare Infection Society. JM has previously received support with conference attendance from Tillott's Pharma. JM chairs the Healthcare Infection Society's Special Interest Group for wastewater and antimicrobial resistance. NS is the supervisor of AKe's Marie Sklowdowska-Curie Actions post-doctoral fellowship (project number: 101203605, project acronym: SELECTION). NS has received support with conference attendance from the Infection Prevention Society (United Kingdom) and European Society for Clinical Microbiology and Infectious Diseases.

All other authors make no further declarations of interest.
